# Optimal Design of Sandwich Composite Cradle for Computed Tomography Instrument by Analyzing the Structural Performance and X-ray Transmission Rate

**DOI:** 10.3390/ma12020286

**Published:** 2019-01-17

**Authors:** Sang Jin Lee, II Sup Chung

**Affiliations:** 1 Composites Convergence Team, Korea Textile Machinery Convergence Research Institute, Gyeongsan 38542, Korea; sjlee@kotmi.re.kr; 2 Department of Mechanical Engineering, Yeungnam University, Gyeongsan 38541, Korea

**Keywords:** computed tomography, sandwich composite, X-ray transmission, CT cradle

## Abstract

Carbon fiber-reinforced composite has an excellent X-ray transmission rate as well as specific modulus and strength. The major components of medical devices, X-ray systems, and computed tomography (CT) equipment that require superior X-ray transmission performance also require structural performance for deformation. Therefore, medical components consist of a sandwich composite structure with carbon fiber composites applied as a face material. The X-ray transmission ratios of face material and foam material were measured according to thickness, and the relation equation for thickness and X-ray transmission rate was derived. The X-ray transmission rate for the sandwich composite structure consisting of face and core material was measured and the relationship between the X-ray transmission rate and the dimension for thickness of sandwich cradle was derived. Using the optimization process, the thicknesses of face and core materials for sandwich cradles were determined to minimize the cost of used materials. They also met the criteria that the deflection should not be more than 20 mm, and the X-ray transmission rate of the cradle should be equal to or greater than that of aluminum at 1.5 mm thickness. The sequence pattern of face material was proposed through structural analysis. The face material of the sandwich cradle was manufactured by a resin infusion and vacuum bagging method, followed by inserting the core between the cured faces. Next, the sandwich cradle assembly was completed and re-cured. The sandwich cradle met the criteria that the deflection at the end was 19.04 mm and the X-ray transmission was 78.7% greater than the X-ray transmission of 1.5 mm aluminum.

## 1. Introduction

Historically, composite materials were lightweight and of superior stiffness and strength. Composite materials were used in aircraft parts and defense components where such characteristics are required [1]. In recent years, their applications have been widely extended to the structures of trains, buses, and other vehicles [2,3,4,5,6,7]. Computed Tomography (CT) is widely used in non-destructive inspections of laminate and sandwich composites [8,9,10,11,12,13,14,15]. Since the early 2000s, composites have been applied to medical device components and are now being used to satisfy X-ray transmission performance and weight requirements. The components of CT and diagnostic X-ray equipment requiring X-ray transmission performance are produced using carbon material. Carbon fiber reinforced plastics (CFRP) components and plates were designed by several optimization processes for the shape and thickness [16,17,18,19,20].

In this paper, the development process of the sandwich cradle for medical devices composed of carbon fiber reinforced plastics CFRP for the face material and foam material as the core was explored. The X-ray transmittance analysis was conducted experimentally to measure the transmission rate according to the thickness of the materials for face material and core material. The specifications were determined to meet the criteria for structural performance and to minimize the cost of applied material for the sandwich cradle. The face material was formed using an infusion and vacuum bagging method. Next, the foam core was inserted between the faces. Finally, the sandwich cradle was assembled and cured to ensure uniform X-ray transmission performance to the X-ray measurement area of the sandwich cradle.

As the purpose of this study, the designing technology of a CFRP sandwich cradle with better X-ray transmission performance than the conventional metallic cradle and the fabricating process of a CFRP sandwich cradle for the uniform X-ray transmission are examined.

## 2. Fundamental Properties of Materials

Among the used fiber materials, CU 125NS (HANKUK CARBON Co., Ltd, Miryang, Korea) and MCU 125NS (HANKUK CARBON Co., Ltd, Miryang, Korea) were unidirectional (UD) prepreg 0.153 mm and 0.155 mm thick, respectively, as shown in Table 1. Plain woven type CF 3327 (HANKUK CARBON Co., Ltd, Miryang, Korea) fabric had a warp density of 11 threads/inch and a weft density of 11 threads/inch. The thickness of CF 3327 is 0.25 mm. CU 125NS was used as the UD carbon fiber, MCU 125 NS was considered to have excellent mechanical properties, and plain-woven fabric CF 3327 was used as the facing material. The core material used was Polymethacrylimide (PMI) foam. Mechanical properties of the materials used are shown in Table 2.

## 3. X-ray Transmission of Materials

### 3.1. Configuration of Test Device

**Figure 1:**
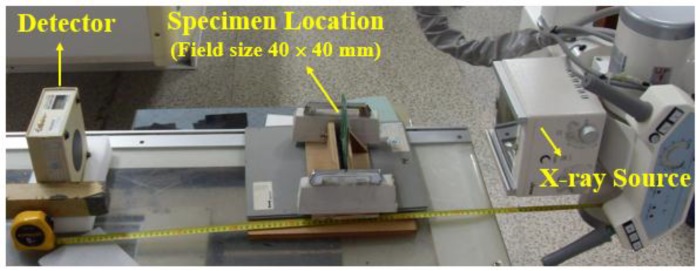
Test configuration of X-ray transmission.

The X-ray transmission equipment used in performance evaluation is a digital X-ray system made by Listem Inc (Wonju-Si, Korea). The configuration of the device for performance evaluation of X-ray transmission is shown in Figure 1. The distance between the source and detector is 1000 mm and the test sample was placed in the center. The field size is 40 mm × 40 mm. The conditions of X-ray transmission were 100 kV, 200 mA, and an exposure time of 0.1 s.

### 3.2. Transmission Performnace of Face materials

**Figure 2:**
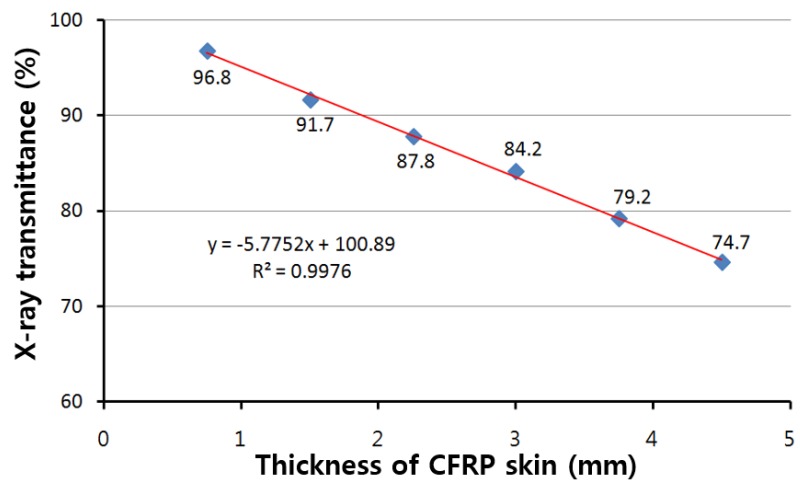
X-ray Transmission ratio of facing materials.

The face materials were chosen from 6 types of UD materials manufactured through vacuum bagging, 2 types of UD + fabric prepreg through vacuum bagging, and 4 types of fabric materials made by infusion as shown in Table 3. The transmission rate was measured as the initial value (I_0_) without specimen applied for 0.1 s at 100 kV, 200 mA and the transmission value (I_1_) measured through each specimen was analyzed under the same conditions. The transmission rate is the ratio of I_1_/I_0_. In the case of the CU 125NS composite, the X-ray transmission rate decreased rapidly from 96.8% to 74.7% as thickness increased from 0.7 mm to 4.5 mm. Regardless of the type of molding technique and material, the transmission rate was approximately the same for the same thickness. Therefore, the relation between the transmission rate and thickness of face material is expressed in the following equation and predicted in Figure 2.

(1) Transmission rate of face material (%)= −5.7752 ×thickness (mm) +100.89

### 3.3. Transmission Performnace of Core Materials

**Figure 3:**
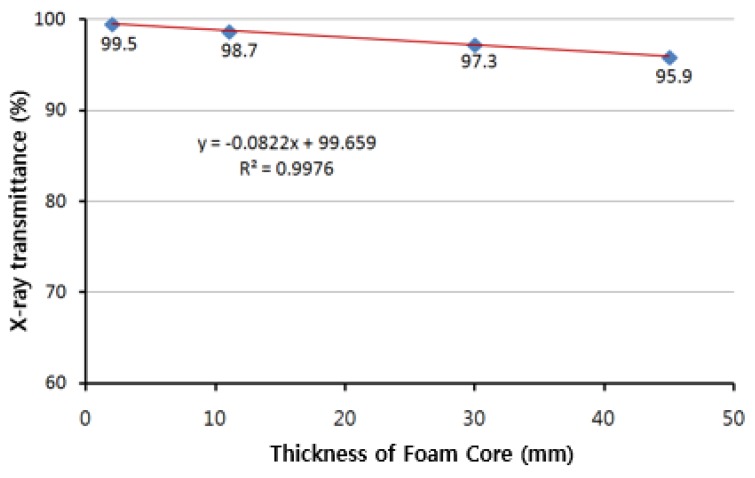
X-ray Transmission ratios of core material.

The X-ray transmission rate of core materials was compared for different thicknesses and densities of foam. These were compared and analyzed for PMI foam 31IG with a density of 32 kg/m^3^ and PVC (Polyvinyl chloride) foam with a density of 40 kg/m^3^. Thicknesses of 2, 11, 30, and 45 mm were considered for PMI Foam 31 IG and an evaluation was performed for PVC foam at thicknesses of 30 and 50 mm. For the PMI 31IG foam, as the thickness of core increased, the transmission rate decreased slightly as shown in Table 4 and Figure 3. PVC foams with a density of 40 kg/m^3^ showed a transmission rate of 90.1% for a thickness of 30 mm when compared to the transmission rate of PMI 31IG with the same thickness. The relationship between the transmission rate and thickness of core material for PMI 31IG is explained in the following equation and predicted in Figure 3.

(2) Transmission rate of core material(%)= −0.0822 ×thickness (mm) +99.659

### 3.4. Transmission Performance of Sandwich Structures

Using the results for X-ray transmission performance of face materials and core materials, several cases of sandwich structure were constructed as shown in the Table 5 and the transmission rate of X-ray was assessed in each case on the same conditions from paragraph 3.1. Through this process, the correlation between the X-ray transmission rate of individual materials and the X-ray transmission rate of sandwich structures was derived. The relationship between the measured value of the actual sandwich structure and the transmission rate calculated using Equation (1) for total thickness of face and Equation (2) for the thickness of core was analyzed in Table 5. The X-ray transmission rate of sandwich structure is estimated by multiplying that of face material calculated by Equation (1) and that of core material calculated by Equation (2). Most cases agree with the following Equation (3) except for some structures made of PVC foam and a thick face.

(3) Transmission rate of sandwich structure(%)=(Transmission rate for total thickness of face calculated from Eq.(1))×(Transmission rate for the thickness of core calculated from Eq.(2))

## 4. Design of CT Cradle

### 4.1. 3-Dimensional Design of Cradle

**Figure 4:**
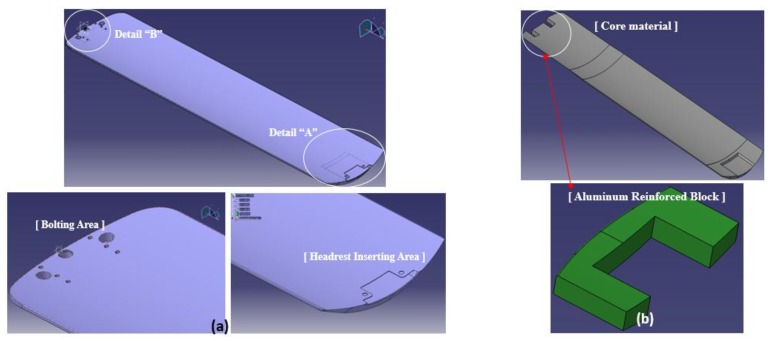
Shape of Sandwich composite Cradle; (**a**) 3-dimensional Shape of Cradle, (**b**) design of Core and the reinforced block.

Figure 4 shows the 3-dimensional shape of the cradle. It consists of the region inserted headrest supported with the patient’s head as shown in detail “A” of Figure 4a and the region fixed at the main frame, as shown in detail “B.” The total length of the cradle is 2322 mm with a width of 465 mm. The core material of the sandwich cradle has the shape inserted in the headrest in the front, as shown Figure 4b, and the shape inserted aluminum reinforcement block for the part bolted through the hole.

### 4.2. Optimization of the Thicknesses of Face/Core Materials

Using the optimization process, the thicknesses of face and core materials for the sandwich cradle were determined to meet the deflection of end point for sandwich cradles were determined not more than 20 mm for the external 135 kg load and the X-ray transmission rate of sandwich cradle was equal to or greater than the X-ray transmission rate of 99.9% aluminum 1.5 mm thickness.

For optimization of the total thickness of the sandwich cradle, the total thickness (x1) of face material and the thickness (x2) of core material were considered as design variables. The objective function is used to determine the cost of material for the sandwich cradle and to determine x1 and x2 to minimize the cost. In general, the price of the face material is 250 US$/(1mm thick) per 1 m^2^ and that of the core material is 1 US$/(1mm thick) per 1 m^2^.

The cost of materials was calculated by:

Area of cradle = Length × width = 2.65 m × 0.5 m = 1.325 m^2^

Cost = f(x1, x2) = (Area × 250 US$) × (x1) + (Area × 1 US$) × (x2)

Therefore, the objective function is described in Equation (4);

(4) Objective function, f(x1, x2)=331.25 ×(x1)+1.325×(x2)

**Figure 5:**
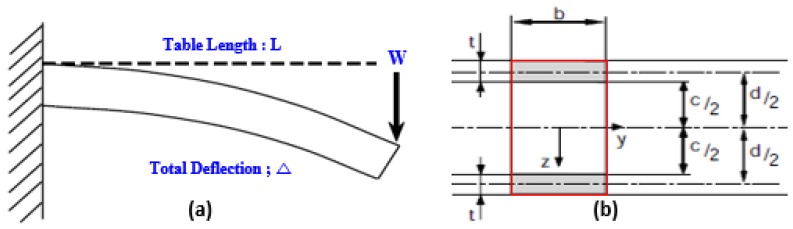
Structural Constraints for sandwich cradle; (**a**) deflection of sandwich cradle for external load, (**b**) cross-section and dimension notation of sandwich beam.

Three conditions were considered to be the constraints for optimization: the limitation of deflection for the end point of sandwich cradle, the condition for X-ray transmission rate of sandwich cradle, and the design limitation for the total thickness of sandwich cradle. At first, the sandwich cradle required that the deflection of the end tip did not exceed 20 mm when a 135 kg load was applied as shown in Figure 5a. The total deflection (Δ) of the sandwich structure is defined in Equation (5) [21]

(5) $$\Delta \;=\;\Delta 1+\;\Delta 2=\;\frac{WL^{3}}{48D}+\;\frac{WL}{4V}$$

where $D=\;E_{f}\frac{bt^{3}}{6}+E_{f}\frac{btd^{2}}{2}+E_{c}\frac{bc^{3}}{12}\;≅\;E_{f}\frac{btd^{2}}{2}$, total thickness of face material x1=2×t, thickness of face material = t, V=AG, A=bd, distance between each center of face material $d=\left(\frac{x1}{2}\right)+x2$, external load W = 135 kg = 1324.35 N, length of cradle L = 2650 mm, width of cradle b = 465 mm, Young’s modulus of face material $E_{f}$ = 50 GPa, Young’s modulus of core material $E_{c}$ = 36.7 MPa, and shear modulus of core material *G* = 6.4 MPa.

Equation (6) represents the first constraint, related to the total deflection and structural requirement.

(6) $$G1\left(x1,\;x2\right)=\;\frac{WL^{3}}{48D}+\;\frac{WL}{4V}-20≦0$$

As the second constraint, the X-ray transmission rate must be greater than that of aluminum 1.5 mm thick (≅ 75.22%).

(7) G2(x1, x2)= 0.7522− A(x1)×B(x2)≦0

where A(x1) and B(x2) were derived from paragraphs 3.2 and 3.3. The relationship between X-ray transmission rate and total thickness of face material is represented by Equation (8).

(8) $$A\left(x1\right)=\;\frac{-5.7752\times x1+100.89}{100}$$

The relationship between X-ray transmission rate and the thickness of core material is represented by Equation (9).

(9) $$B\left(x2\right)=\;\frac{-0.0822\times x2+99.659}{100}$$

The third constraint is the limitation of the total thickness (T) of the sandwich cradle. Total thickness of the sandwich cradle must not exceed 50 mm. Therefore, this constraint is expressed by Equation (10).

(10) G3(x1, x2)= x1+x2−50≦0

**Figure 6:**
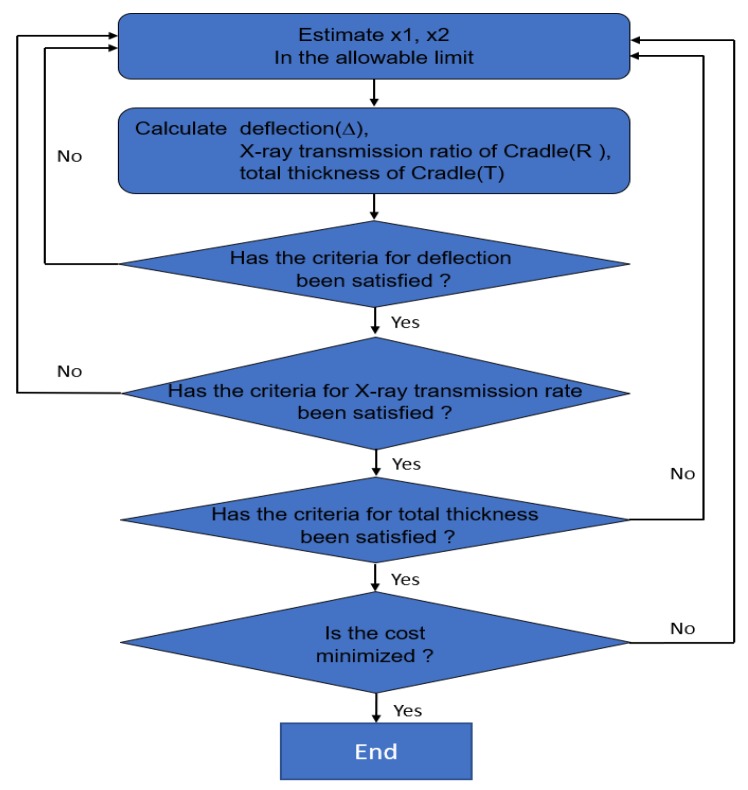
Optimization Algorithm for Sandwich Cradle.

The optimization solution used in this study has been applied with linear programming (LP) using Microsoft Excel (Microsoft Corporation, Redmond, WA, USA). As shown in Figure 6, the optimization algorithm (1) suggests values for x1 and x2, (2) calculates deflection, X-ray transmission rate of sandwich cradle, and the total thickness of sandwich cradle, (3) repeats the process of verifying that the calculated values are met with constraint conditions, and (4) determines the x1 and x2 values with minimum material cost. The resulting total thickness (x1) of face and thickness (x2) of the core was decided to be 2.40 and 47.60 mm, respectively.

### 4.3. Prediction of Stacking Sequences

**Figure 7:**
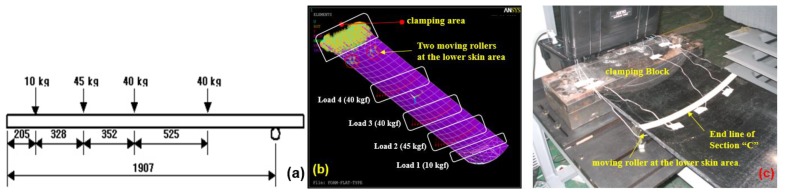
Loading conditions and Boundary conditions on structural analysis; (**a**) Loading conditions on the top surface, (**b**) Loading & Boundary conditions on ANSYS, (**c**) Real clamping condition.

The structural performance criteria of the cradle that the end point deflection should not exceed 20 mm when the total load applied at a specific location is 135 kg is shown in Figure 7a. Figure 7b,c indicate the boundary conditions of structural analysis. The finite element analysis (FEA) for prediction of the stacking sequence was performed using ANSYS Version 11 (Taesung S&E, Seoul, Korea). Using 3-D CAD data, the core was modeled by a solid element (Solid 95) and the facing materials were applied with layered shell elements (Shell 99). Each attribution, such as the proposed stacking patterns, material property, thickness, and stacking direction, were assigned to each portion of the facing material.

**Figure 8:**
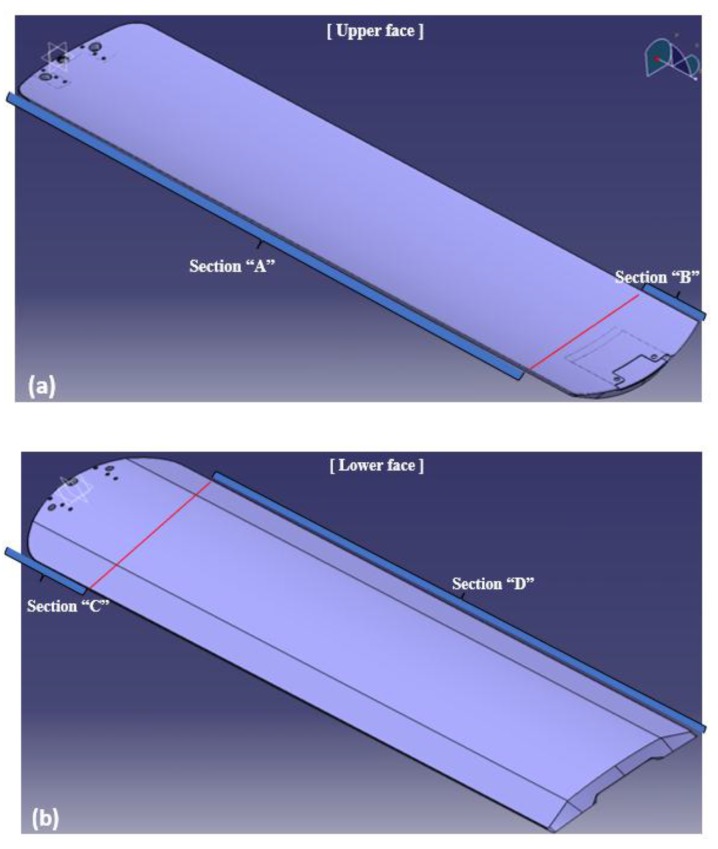
Proposed Sequence pattern; (**a**) Upper face (**b**) Lower face.

**Figure 9:**
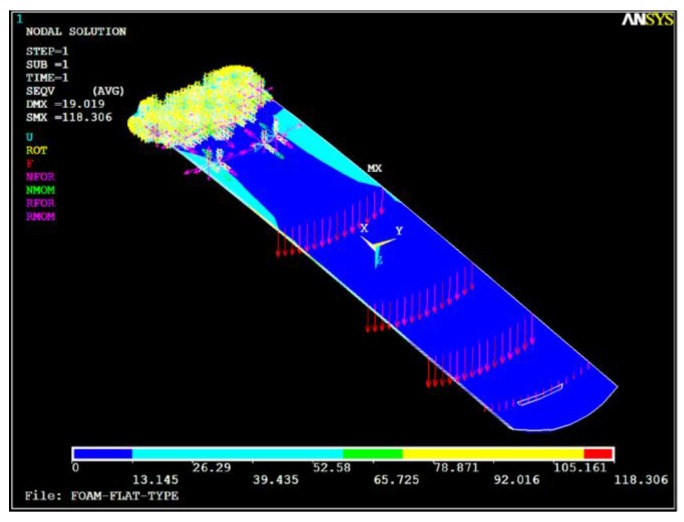
Result for structural analysis of sandwich cradle.

In Figure 8, the proposed stacking pattern is that CF 3327 (4 plies) is applied on the upper skin (Section A) and reinforced CF 3327 (2 plies) is applied in to the Section B area. CF 3327 (3 plies), MCU 125NS (3 plies) and CU 125 NS (3 plies) were proposed to be applied at Section D and were reinforced with CF 3327 (9 plies), MCU 125NS (3 plies), and CU 125 NS (19 plies), for a total of 31 plies at Section C for the lower skin. For all materials, the fiber orientation was laminated at 0 degrees. As the result of FEA, the end deflection was estimated as 19.02 mm, which satisfies the requirements as shown in Figure 9. As the result of FEA, the thickness of the X-ray transmission area was 2.65 mm and was similar to the thickness of the optimization results.

## 5. Manufacturing Process and Performance Evaluation of CT Cradle

### 5.1. Manufacturing Process of Cradle

**Figure 10:**
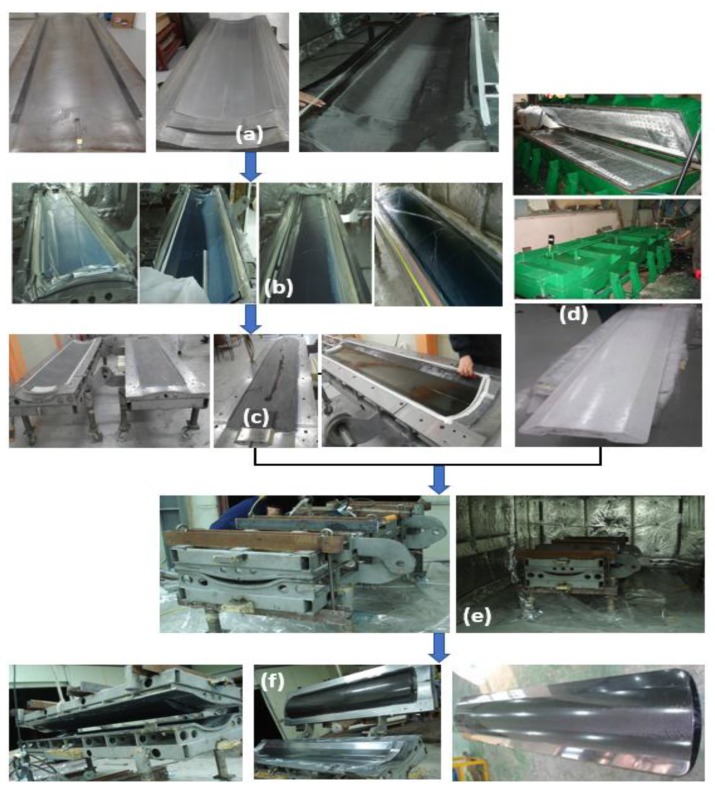
Manufacturing Process of Sandwich cradle; (**a**) preparing mold & layup of carbon fiber, (**b**) resin infusion, (**c**) curing the faces & apply the resin evenly, (**d**) prepared core, (**e**) inserting core into molds and curing/bonding, (**f**) de-molding and hole machining.

The cradle designed as a sandwich structure is composed of the top face, bottom face, and the foam core between faces. The first layers of upper and lower face were formed by infusion and the remaining layers were formed using prepreg. The upper and lower faces were molded as shown in Figure 10a,b, after stacking carbon UD or carbon fabric as per the thickness estimated by (FEA). After applying additional resin on the cured face, the prepared core was inserted between two molds as shown in Figure 10c,d, and it was assembled by clamping. Then, the assembly was cured in a dry oven as shown in Figure 10e. After curing, the assembly was demolded, and the completed cradle was trimmed at the bonded area as shown in Figure 10f.

### 5.2. Stiffness Evaluation of Cradle

**Figure 11:**
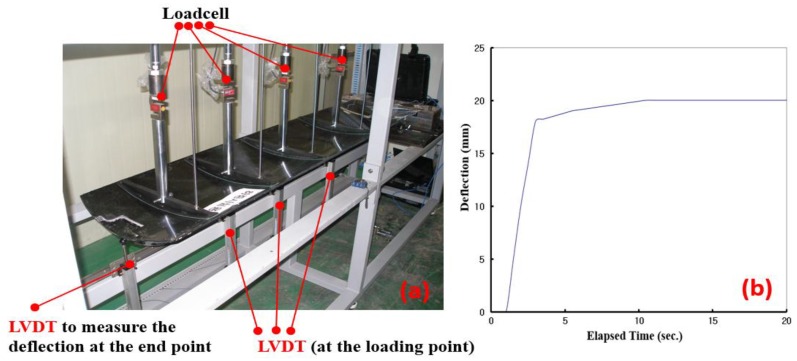
Real deflection test; (**a**) Test set-up, (**b**) Result of deflection test.

A stiffness test of the cradle determined whether it satisfied the criteria, which is that the end deflection is 20 mm or less when a total load of 135 kg was applied. The loading bar connected to the loadcell and LVDT (Linear Variable Differential Transformer) were equipped and the applied load and deflection at the prescribed point were measured as shown in Figure 11. As a result, the deflection at the end point of the cradle was found to be 19.49 mm. Therefore, it satisfied the criteria.

### 5.3. X-ray Transmission Performance of Cradle

**Figure 12:**
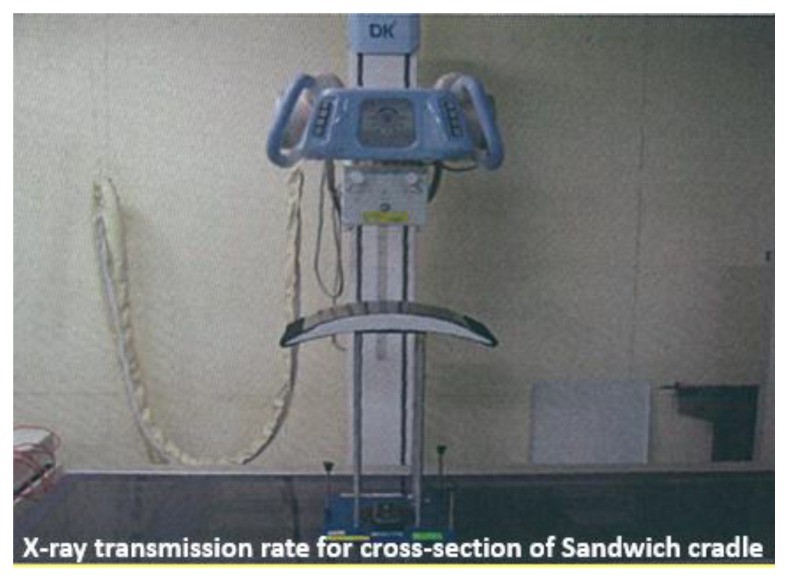
X-ray transmission test for thick section of sandwich cradle.

The X-ray transmission rate for the final product was measured as shown in Figure 12 for the thickest part of the X-ray measurement area of the sandwich cradle. The conditions of X-ray transmission were 100 kV, 200 mA, and an exposure time of 0.1 s. The transmission rate of the sandwich cradle was 78.8%, which is better than the 75.22% transmission rate of the 1.5 mm thick aluminum (Table 6).

**Figure 13:**
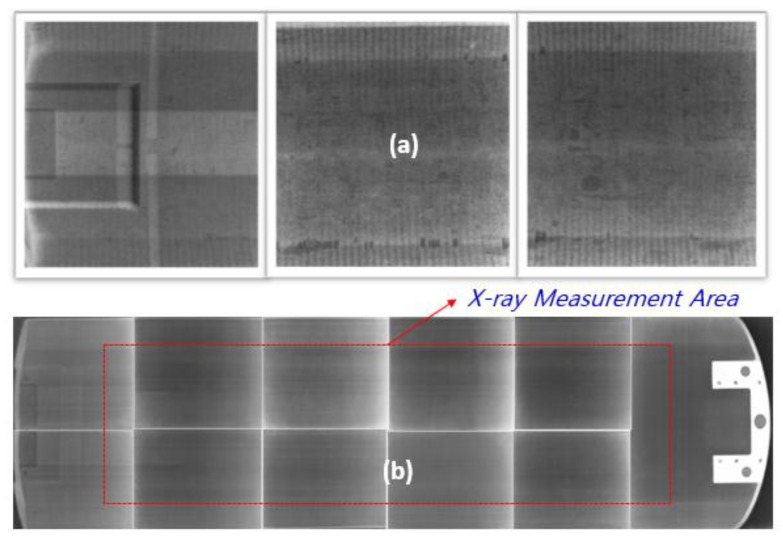
Uniform X-ray transmission of Composite Cradle; (**a**) bad case due to the void, excess resin, and poor resin of face and wrinkle of foam, (**b**) good case - 11 spit-shots of X-ray measurement area.

Uniform X-ray transmission performance of the sandwich cradle whose first ply for the upper face and lower faces was laid by infusion and the remaining layers used prepreg is shown in Figure 13b. This is in contrast to the usual defect as shown in Figure 13a.

## 6. Conclusions

This paper evaluated the X-ray transmission rate for face, core and sandwich structure to determine the thickness of the sandwich cradle by repeatedly calculating the thickness of face and core materials to satisfy the structural performance, X-ray transmission conditions, and to propose the manufacturing process for the sandwich cradle.

1. The X-ray transmission performance of several face materials and some cases of core materials was measured. Measurements of the X-ray transmission rate of individual materials show that X-ray transmission performance was significantly affected by the density and thickness of the materials. The equations describing the relationship between thickness of face and core material and the X-ray transmission rate were derived through the measured value. X-ray transmission rates were measured for a sandwich structure combined with several cases of face and core, and the relation equation for X-ray transmission performance of sandwich structure was estimated.
2. The 3D shape of the sandwich cradle of CT instruments was decided with consideration to the bolting conditions and interface with other parts. The thicknesses of face and core materials were optimized to minimize the cost of the materials on meeting the requirements that the deflection of sandwich cradle should be not more than 20 mm and the X-ray transmission rate of cradle should be equal to or greater than that of aluminum 1.5 mm thick.
3. The first layers of upper and lower skin were formed by infusion and the remaining layers were used prepreg. After the upper and lower skins were molded, they were integrally bonded to the core and the sandwich cradle was formed. X-ray transmission performance of the final fabricated cradle was uniform over its entire surface. The X-ray transmission rate of the sandwich cradle is 78.8%, which is better than the 75.22% transmission rate of aluminum (1.5 mm thick). Additionally, it satisfied the criteria by finding the deflection at the end point of the cradle to be 19.49 mm.
4. The improvement of the CT cradle increases the accuracy of the medical device rather than the improvement of the CT system and an improvement in X-ray transmission rates can have an effect on reducing the amount of X-ray applied to patients.

## Figures and Tables

**Table 1:** Specifications of applied fiber.

Material	Carbon Fiber Weight (g/m^2^)	Resin Weight (g/m^2^)	Resin Contents (%)	Thickness (mm)
CU 125NS Prepreg *	125	62	33	0.153
MCU 125NS Prepreg *	125	64	34	0.155
CF 3327 **	205	105	33.8	0.25

* The density of CU 125NS (Carbon Uni-directional, 125 g/m^2^, No Scrim) composite cured by vacuum bagging is 1500 kg/m^3^. The density of MCU 125NS (Medium modulus Carbon Uni-directional, 125 g/m^2^, No-Scrim) is 1500 kg/m^3^. ** CF 3327 (Carbon Fabric) composite was fabricated by infusion and the density was similar to 1500 kg/m^3^.

**Table 2:** Mechanical properties of applied materials.

Property Material	Tensile Modulus (GPa)	Shear Modulus (GPa)	Poisson’s Ratio	Strength (MPa)
CU 125NS	E_1_ = 127.6 E_2_ = 7.58	G_12_ = 4.05	V_12_ = 0.34 V_21_ = 0.05	2650 65.5
MCU 125NS	E_1_ = 191.4 E_2_ = 8.58	G_12_ = 5.10	V_12_ = 0.24 V_21_ = 0.04	-
CF 3327	E_1_ = 48.3	G_12_ = 3.81	V_12_ = 0.07	548.9
PMI foam (31 IG)	0.036	0.013	0.02	1.0

**Table 3:** X-ray Transmission ratio of CFRP.

Face material	**Fabrication**	**Material of Face**	**Total Thickness (mm)**	**Measured Value (I_1_) (unit: mRem)**	Ratio of Transmission (I_1_/I_0_ *) × 100	**Remark**
	Prepreg vacuum bagging	100% CU 125NS	0.75	214	96.8	A(1)
		100% CU 125NS	1.5	203	91.9	A(2)
		100% CU 125NS	2.25	194	87.8	A(3)
		100% CU 125NS	3	186	84.2	A(4)
		100% CU 125NS	3.75	175	79.2	-
		100% CU 125NS	4.5	165	74.7	-
	Prepreg vacuum bagging	33.3% CU 125NS + 66.7% CF 3327	2.25	194	87.8	B(1)
		60% CU 125NS + 40% CF 3327	3.75	175	79.2	B(2)
	Infusion	100% CF 3327 fabric	0.75	213	96.4	-
		100% CF 3327 fabric	1.5	202	91.4	C(1)
		100% CF 3327 fabric	3	185	83.7	C(2)
		100% CF 3327 fabric	4.5	165	74.7	C(3)

* The value of I_0_ is 221. The densities of above composites were controlled as 1500 kg/m^3^.

**Table 4:** X-ray Transmission ratio of foam material.

Core	**Material**	**Density (kg/m^3^)**	**Total Thickness (mm)**	**Measured Value (unit: mRem)**		**Ratio of Transmission (I_1_/I_0_) × 100**
				**I_0_**	**I_1_**	
	PMI foam, 31 IG	32	2	221	220	99.5
			11	223	220	98.7
			30	223	217	97.3
			45	223	214	95.9
	PMI foam, 51 IG	51	45	223	208	93.3
			60	223	203	91.2
	PVC foam	50	45	223	209	93.7
			60	223	204	91.5

**Table 5:** X-ray Transmission Ratios for Sandwich Structure.

Sandwich Structure * (face/core/face)	Total Thickness (mm)	Measured Value (unit: mRem)		Ratio of Transmission (I_1_/I_0_) × 100	Transmission Rate of Face Calculated From Eq.(1)	Transmission Rate of Core Calculated From Eq.(2)
		I_0_	I_1_			
A(1) + PMI(31 IG, 11t) + A(1)	12.5	223	192	86.1	92.2	98.7
A(2) + PMI(31 IG, 11t) + A(2)	14	223	178	79.8	83.5	98.7
A(1) + PMI(51 IG, 45t) + A(1)	46.5	223	193	86.5	92.2	96.0
A(2) + PMI(51 IG, 45t) + A(2)	48	223	177	79.4	83.6	96.0
A(2) + PVC(30t) + A(2)	33	220	164	74.5	83.6	97.2
A(2) + PVC(50t) + A(2)	53	220	163	74.1	83.6	95.5
B(1) + PMI(51 IG, 45t) + B(1)	49.5	220	160	72.7	74.9	96.0
B(2) + PVC(30t) + B(2)	37.5	222	144	64.9	57.6	97.2
B(2) + PVC(50t) + B(2)	57.5	222	143	64.4	57.6	95.5
C(1) + PMI(31 IG, 11t) + C(1)	14	222	178	80.2	83.6	98.7
C(2) + PMI(31 IG, 11t) + C(2)	17	222	166	74.8	66.2	98.7
C(1) + PMI(51 IG, 45t) + C(1)	48	222	177	79.7	83.6	96.0
C(2) + PMI(51 IG, 45t) + C(2)	51	222	160	72.1	66.2	96.0
C(2) + PVC(30t) + C(2)	36	222	150	67.6	66.2	97.2
C(2) + PVC(50t) + C(2)	56	222	148	66.7	66.2	95.5
C(1) + PMI(31 IG, 45t) + A(3)	48.75	222	172	77.5	79.2	96.0
C(1) + PVC(50t) + A(3)	53.75	222	159	71.6	79.2	95.5
C(2) + PMI(51 IG, 45t) + A(2)	49.5	222	168	75.7	74.9	96.0
C(2) + PVC(50t) + A(2)	54.5	222	156	70.3	74.9	95.5
B(1) + PMI(51 IG, 45t) + B(2)	51	222	160	72.1	66.2	96.0
B(1) + PVC(50t) + B(2)	56	222	149	67.1	66.2	95.5

* Detail Specifications of Materials are; refer to the Table 3 and Table 4. In Table 3/Table 4, A(1) CU 125NS prepreg, Vacuum bagging, 0.75 mm thick; A(2) CU 125NS prepreg, Vacuum bagging, 1.5 mm thick; A(3) CU 125NS prepreg, Vacuum bagging, 2.25 mm thick; B(1) CU 125NS(0.75 t) + CF 3327(1.5 t), Vacuum bagging, 2.25 mm thick; B(2) CU 125NS(2.25 t) + CF 3327(1.5 t), Vacuum bagging, 3.75 mm thick; C(1) CF 3327 fabric(6 plies), Infusion, 1.5 mm thick; C(2) CF 3327 fabric(12 plies), Infusion, 3.0 mm thick; C(3); CF 3327 fabric(18 plies), Infusion, 4.5 mm thick.

**Table 6:** X-ray Transmission rate of Final sandwich Cradle.

	Measured Value (unit: mRem)				X-ray Transmission Rate (%) (I1/I0) × 100
	1	2	3	Average	
I_0_ (Initial value)	222.4	219.2	218.2	220.0	78.8
I_1_ (sandwich cradle)	174.6	172.4	172.6	173.2	
